# Detergent-based separation of microbes from marine particles

**DOI:** 10.1128/aem.01426-25

**Published:** 2025-09-25

**Authors:** Jordan T. Coelho, Lauren Teubner, J. Cameron Thrash

**Affiliations:** 1 Department of Biological Sciences, University of Southern California 118558, Los Angeles, California, USA; Indiana University Bloomington, Bloomington, Indiana, USA

**Keywords:** detergents, particle-associated microbes, marine particles

## Abstract

Marine particles, typically composed of organic detritus and cellular debris, harbor microbial communities that are distinct from the planktonic, or free-living, communities in the pelagic ocean. However, without being first separated from the particle and microbial consortia, these microbes are inaccessible to further investigation via single-cell microbiology methods like flow cytometry, cell sorting, and dilution-based isolation. To confront this obstacle, we compared the dissociative effects of two commonly used detergents, Tween 20 and Tween 80, on particle-associated marine microbial communities. The ability of Tween treatments to liberate cells from particles and to maintain cell integrity was quantified by flow cytometry from multiple communities across seasons and locations. Both Tween 20 and Tween 80, at 185 RPM shaking, gently dissociated microbes from their particles, causing very little cell mortality. Additionally, Tween 80 liberated a greater number of particle-associated cells into the free-living fraction. We also analyzed the effects of Tween treatments on the microbial community composition for one of these collections via 16S rRNA gene amplicon sequencing of the particle-associated and free-living fractions relative to unamended controls. Tween 20 and Tween 80 were both effective for microbial detachment from particles; however, Tween 80 treatments displayed greater uniformity in the dissociated communities and significantly enriched for the most abundant particle-associated members. Together, these data indicate that Tween 80 was most effective at gently dissociating particle-associated cells.

**IMPORTANCE:**

Microbes that reside on marine particulate organic matter are vital facets of marine biogeochemistry. As they degrade the particle on which they reside, the resulting concentrated region of activity influences surrounding biogeochemistry and redox gradients, making particle-associated microbes significant to overall marine ecology. To understand single-cell activities amidst the microbial assemblage on the particle, cells must first be removed from the substrate for downstream analyses. Methods for microbial dissociation from solid surfaces or sediment communities have been described; however, analogous methods for more ephemeral particles that also maintain cell viability and preserve DNA for next-generation sequencing are understudied. Here, we optimized a method that leveraged detergents to dissociate microbes from marine particles. We evaluated effectiveness through filter size fractionation, flow cytometry, and community composition analyses and provided recommendations to gently and effectively remove microbes from marine particles.

## INTRODUCTION

Marine particles result from a wide range of biotic and abiotic sources (1) and are largely composed of aggregates of cellular debris, fecal pellets, and organic detritus (2). These particles are broadly defined as particulate organic matter (POM) and, due to their concentration of nutrients and carbon, represent hotspots of microbial activity where microbes transiently or permanently reside (3–5). Particle-associated (PA) microbes form complex communities that coordinate their efforts to degrade the POM (6, 7), and often the PA microbial assemblage reflects the array of metabolic niches present within the particle (8–10). PA communities are taxonomically distinct from planktonic/free-living (FL) communities and can be influenced by differing environmental conditions, with genomic evidence suggesting strikingly different physiologies between the two communities (11–14). Thus, PA communities occupy separate ecological niches than those of FL communities. However, PA communities are generally inaccessible to single-cell microbiology methods (15), including flow cytometry and fluorescence-activated cell sorting, dilution-to-extinction cultivation methods, and single-cell genomics, all of which have greatly contributed to our understanding of the ecophysiology of many important microbial lineages and ecological processes (16–22). For example, PA microbes live in dense consortia inside or on the surface of particles, so a flow cytometer cannot distinguish individual cells, and particles may obstruct the fluidic lines of the instrument. Other single-cell methods face similar challenges. Thus, dissociation of PA microbes from their particles makes them available for a wider array of methods to investigate their ecophysiologies.

Many methods have been developed to separate microbes from surfaces. An effective approach for detaching particle-associated and sediment-associated microbes involved 10% (vol/vol) methanol and sonication (23). Although the methanol disruption approach has been applied across marine particles (24), ruminal digesta solids (25), and deep-sea sediments (26), 10% (vol/vol) methanol requires a large chemical addition to the sample and is toxic to microbial cells (27), thus inhibiting growth and propagation of the dissociated cells. Other methods using low pH, formaldehyde, tertiary butanol, and methylcellulose (25, 28) have similar toxic effects on microbial cells, and some also prevent downstream DNA sequencing (29). Density centrifugation is another method that has been successful in detaching sediment-associated cells; however, the protocol is lengthy, the reagents are expensive, and the multiple centrifugation and supernatant removal steps can lead to cell losses (26, 30).

Detergents are a popular choice for dissociating microbes from sediments (31), soils (32), surfaces in the food industry (33, 34), and atmospheric particles (35) because they disrupt the linkages between the microbe and the particle (35). Among the commonly used detergents are pyrophosphate and Tween, representing ionic and nonionic detergents, respectively. Ionic detergents contain a charged polar head group that elicits a strong destabilizing effect, making them ideal constituents in cell lysis buffers (36, 37). However, if the goal is to maintain cell membrane integrity and viability, ionic detergents are not the optimal choice due to their high affinity for proteins (38) and lipid membranes (39). Nonionic detergents, with an uncharged polar head group, are a gentler option for disrupting linkages between microbes and particles. Furthermore, the ionic strength of the surrounding matrix has little to no effect on the efficacy of nonionic detergents (40), making them a suitable choice for marine applications.

The Tween family of nonionic detergents is frequently used for stabilizing enzymes (41) and isolating proteins (42), and dilute concentrations have previously been used for dissociating microbes from sediments and atmospheric particles for downstream flow cytometry applications (30, 31, 35). Tween 20 and Tween 80 are commonly used Tween detergents; however, their efficacy has not yet been proven for the removal of microbes from marine particles. The same concentrations and variations of these detergents that have been previously proven effective on sediment (30, 31) or atmospheric (35) microbial communities, among others, may not have the same effect on marine PA communities because of the fragile and ephemeral nature of marine particles, the distinct and dynamic communities, processes, and interactions occurring on them (6, 43, 44), and the multiple mechanisms that microbes use for attachment (45).

We tested the efficacy of Tween 20 and Tween 80 for liberating cells from marine particles. We quantified the effectiveness of Tween treatments in samples from different seasons and locations using flow cytometry to enumerate the number of viable cells dissociating from the PA fraction into the FL fraction and analyzed the PA and FL microbial community composition via 16S rRNA gene amplicons after Tween treatments relative to unamended controls to determine which organisms were being affected by the treatment. Tween 80 treatments consistently enriched for the most abundant particle-associated taxa, whereas Tween 20 treatments were more variable and enriched for rarer particle-associated taxa, corroborating flow cytometry observations that low concentrations of Tween 80 were the most effective for dissociating PA microbes from marine particles.

## RESULTS AND DISCUSSION

### Experimental design

**Fig 1:**
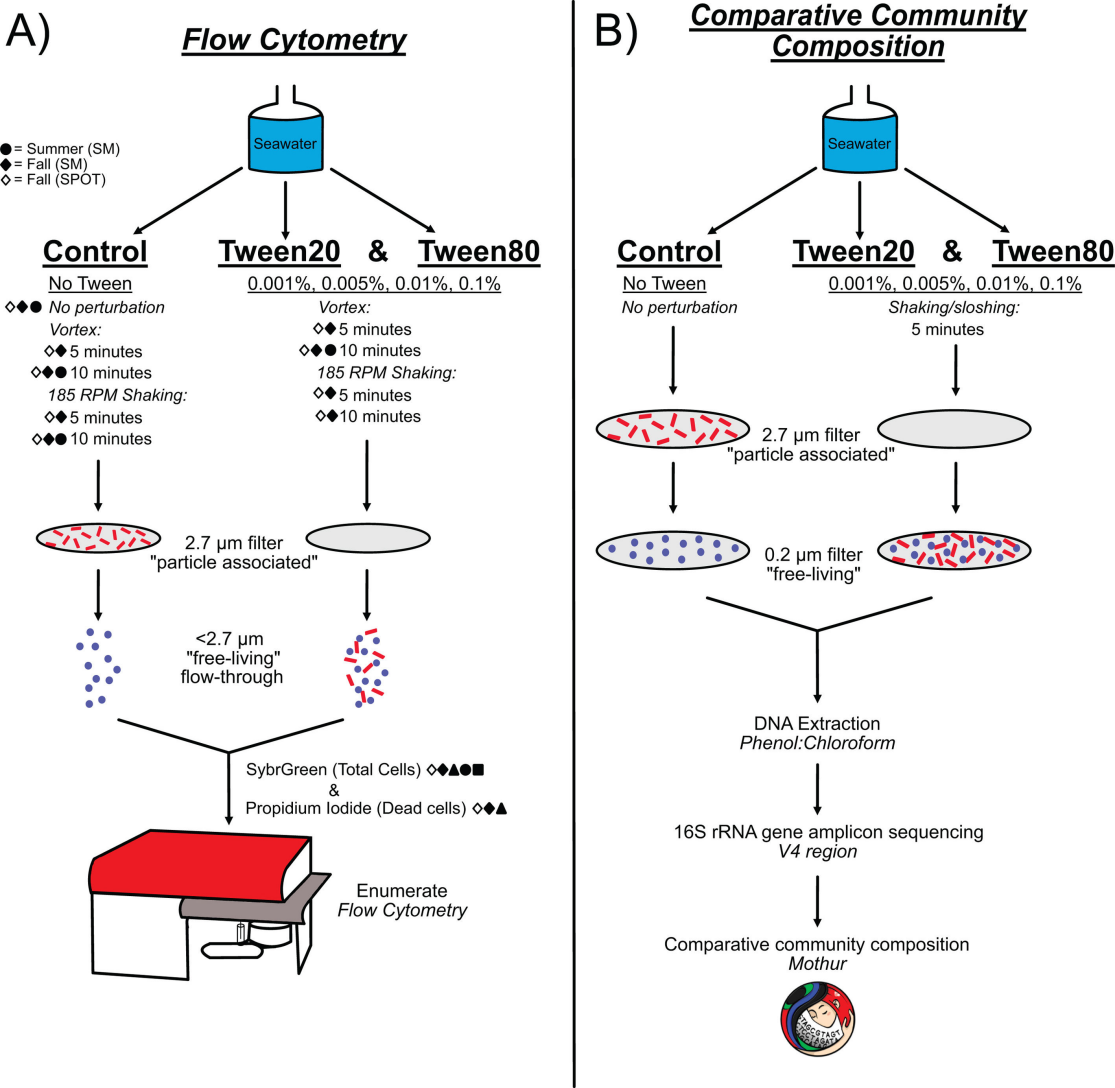
Experimental design and workflow. Experimental conditions are described with underlined text, and the perturbation method is described by italicized text with the duration of perturbation listed below. The filter size fractionation of the particle-associated community (red) and the free-living community (blue) is depicted by the gray discs that represent the filters, with the particle-associated community becoming enriched in the planktonic size fraction after Tween treatments. (**A**) Experimental design and workflow for the verification of microbial dissociation from particles via flow cytometry. Not every seawater collection received the same perturbation methods and durations; therefore, the shapes indicate the season and location from which seawater was collected for experimentation: Summer, Santa Monica (filled circle); Fall, Santa Monica (filled diamond); and Fall, the San Pedro Ocean Time series station (SPOT) (open diamond). (**B**) Experimental design and workflow for the verification of microbial dissociation from particles via 16S rRNA gene community composition analysis.

We developed an experimental plan based on a common sequential filtration procedure to separate particle-associated from free-living marine microorganisms. We chose 2.7 µm as a cutoff to define PA communities and 0.22 µm to capture most remaining cells (Fig. 1). Our experimental logic was that an effective dissociative treatment that liberated cells from particles would result in a greater proportion and diversity of microbes passing through the 2.7 µm filter than without such a treatment. Our treatments consisted of the commonly used detergents, Tween 20 and Tween 80, which we tested at four concentrations spanning previous ranges used for liberation of cells from sediments (0.001%, 0.005%, 0.01%, and 0.1% [vol/vol]), along with controls that did not receive Tween. We then used flow cytometry to measure changes in bulk cell numbers and viable cells across multiple treatment types and samples because it is high-throughput, requires low sample volumes, and also provides greater accuracy than direct microscopic counts (46) (Fig. 1A). Furthermore, we expected our treatments to degrade some particles completely, like common ephemeral gel-like substrates, and thus make microscopic evaluation of the change in the number of cells on particles unreliable. We chose to use propidium iodide as a proxy for cell viability since it could be used in the high-throughput context of flow cytometry and avoids culture biases associated with viability measurements like colony-forming units (47–49). Additionally, we performed community composition analyses via 16S rRNA gene amplicon sequencing to compare the differences in microbial cells passing through the 2.7 µm filter after Tween treatments and identify which taxa were liberated from the particles (Fig. 1B).

### Cellular dissociation and viability

**Fig 2:**
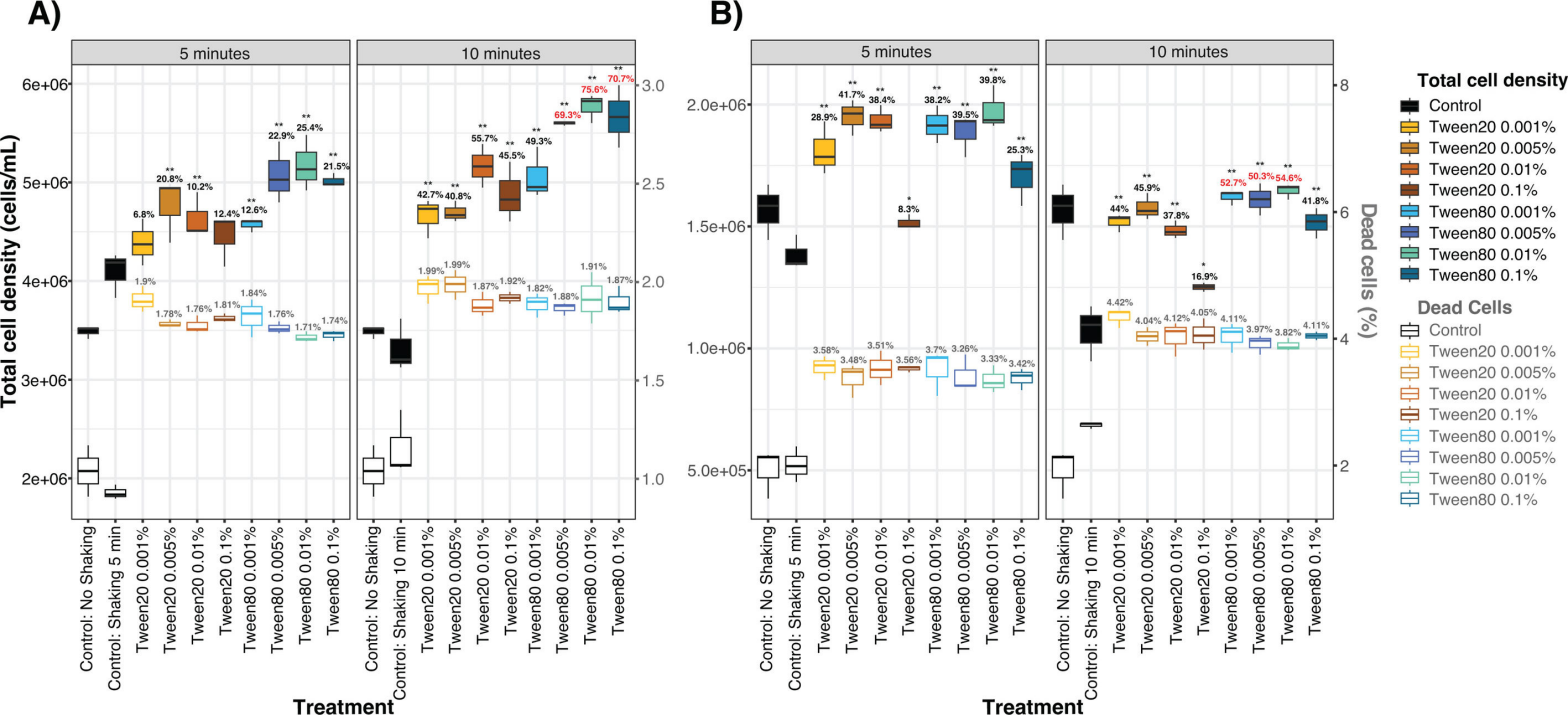
Microbial dissociation from marine particles evaluated by flow cytometry for the Santa Monica Bay (**A**) and SPOT (**B**). *X*-axes indicate treatments. The left *y*-axis displays the total cell density because of the SYBR Green fluorescent stain (black text), and the right *y*-axis displays the cell density of the compromised cells within the sample because of the propidium iodide fluorescent stain (gray text). The data are separated by the duration of perturbation, either 5 or 10 minutes of 185 RPM shaking. The boxplots show the lower and upper quartiles with the horizontal line, indicating the median and the whiskers showing the minimum and maximum values. The percentages above the boxplots indicate the average percent increase in cell density or percent cell death relative to the control that received physical perturbation. The top three most successful Tween conditions that dissociated the most particle-associated cells are colored in red. Asterisks above the boxplots and percentage values indicate the statistical significance and amount of increase in cell density relative to the control that received physical perturbation, respectively. *P value = 0.05 and **P value < 0.05.

We evaluated the dissociation efficiency of Tween treatments via flow cytometry at two different sampling sites: Santa Monica Bay, to represent a coastal system, and the San Pedro Ocean Time series station (SPOT), to represent an open ocean system. We quantified total FL cells and FL cells with compromised membranes after 5 or 10 minutes of shaking at 185 RPM relative to no-Tween shaken controls and no treatment controls (Fig. 2). In our Fall 2023 Santa Monica Bay experiment, we observed the greatest liberation of cells with 10 minutes of shaking in Tween 80 0.005%, 0.01%, and 0.1%, yielding median percent increases in cell density from the control of 69.3%, 75.6%, and 70.7%, respectively (Fig. 2A). Cell mortality was similar across all Tween/shaking treatments at below 2% of the total cell count. Controls showed roughly 1% cell mortality. Our Spring 2024 SPOT experiment had different absolute numbers, but the trends corroborated our previous experiment. Tween 20 and Tween 80 had more similar results at 5 minutes of shaking, but at 10 minutes of shaking, Tween 80 yielded slightly greater cell dissociation, based on the percent cell increases relative to controls (Fig. 2B), though nearly all Tween treatments resulted in significant increases (*P* value ≤ 0.05) in FL cell density compared to the control treatment that received physical perturbation (Table S1). We observed the highest dissociation with 10-minute shaking in Tween 80 0.001%, 0.005%, and 0.01%, with median percent increases in cell density from the control of 52.7%, 50.3%, and 54.6%. Cell mortality was similar between both Tween 20 and Tween 80 and represented less than 4.5% of the total cell count, compared to ~2%–3% mortality in the controls. The higher mortality observed in the SPOT samples compared to Santa Monica Bay could have resulted from differences in sample collection and processing times (see Materials and Methods); however, both experiments indicated that 10-minute shaking with Tween 80 at 0.005%–0.01% dissociated the most PA cells into the FL fraction.

Variations to the perturbation type and duration confirmed Tween 80 and 10 minutes of 185 RPM shaking to be the most consistent treatments. Experiments using Santa Monica Bay surface water across multiple seasons showed that vortexing introduced more variability in dissociation frequency, and in some cases, drastically increased cell mortality, compared with 185 RPM shaking (Fig. S1). Direct comparison of 10-minute shaking vs vortexing using a summer surface sample showed comparable results (Fig. S1A). However, when comparing 5 vs 10-minute vortexing with a fall sample, we found very high cell mortality in the 10-minute treatment, between 15% and 17%, far exceeding the 2%–3% of the untreated controls (Fig. S1B). Comparing cell mortality after 10-minute vortexing in the summer (Fig. S1A) vs the fall (Fig. S1B) suggested that vortexing differentially affected cell mortality depending on the presumed variation in microbial community composition between seasons. Shaking at 185 RPM provided comparable or higher dissociation of cells than vortexing and with more consistent outcomes in cell mortality across summer and fall seasons (Fig. 1; Fig. S2A). Collectively, our experiments indicated that Tween 80 with gentle shaking for 10 minutes reliably dissociated PA cells from a variety of different sample types and frequently at higher numbers than Tween 20.

### Community effects

**Fig 3:**
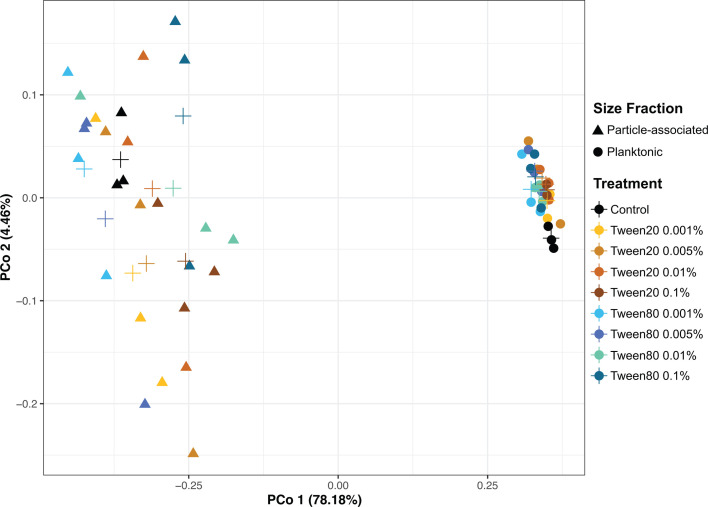
Principal coordinate analysis of the particle-associated and free-living communities in response to Tween treatments. Shapes on the plot indicate either particle-associated (triangle) or free-living (circle) communities, and the color of the shapes corresponds to the experimental condition or control, according to the key. The crosses indicate the centroid points for each treatment.

To evaluate the effect of the dissociation treatments on the microbial community and to identify which taxa were liberated from particles, we used whole community 16S rRNA gene amplicon sequencing of communities obtained from surface water, treated with the same suite of Tween conditions and gentle manual shaking of carboys (roughly equivalent to 185 RPM shaking) described above. As expected, based on many prior observations (12, 50–53), the PA and FL communities were distinct as evaluated by principal coordinate analysis (PCoA), with PCo1 separating the PA and FL communities and explaining 78.18% of variance (*R^2^* = 0.96) (Fig. 3). PCo2, which appeared related to the Tween treatments, explained only 4.46% of variance (*R^2^* = 0.98). In both the PA and FL communities, the community structure diverged away from the tightly clustered control communities after Tween treatments. We also observed a slight movement in the FL communities along PCo1 after treatment, suggesting that some liberated PA taxa were affecting the composition of the FL community. Replicates of PA communities were highly divergent and did not form consistent clusters based on Tween treatments, suggesting a high degree of heterogeneity in the dissociative results. To quantify the degree of heterogeneity across treatments, we examined the percentage of operational taxonomic units (OTUs) that were shared or unique among the replicates in each of the treatments (Fig. S2). An average of 43% of OTUs were unique to a single replicate across both the PA and the FL communities, indicating considerable variation in the microbial communities collected across filters and treatments and corroborating the observed variance in PCo2 across treatments. We detected an average of 37% of the OTUs across all three replicates in both the control PA and control FL treatments. Therefore, we separately defined PA-OTUs and FL-OTUs as those that were detected across all three replicates in control 2.7 and 0.22 µm filters, respectively, and then tracked changes of these “core” OTUs to gain a meaningful understanding of how their relative abundances changed in response to Tween treatments.

### OTU specificity

**Fig 4:**
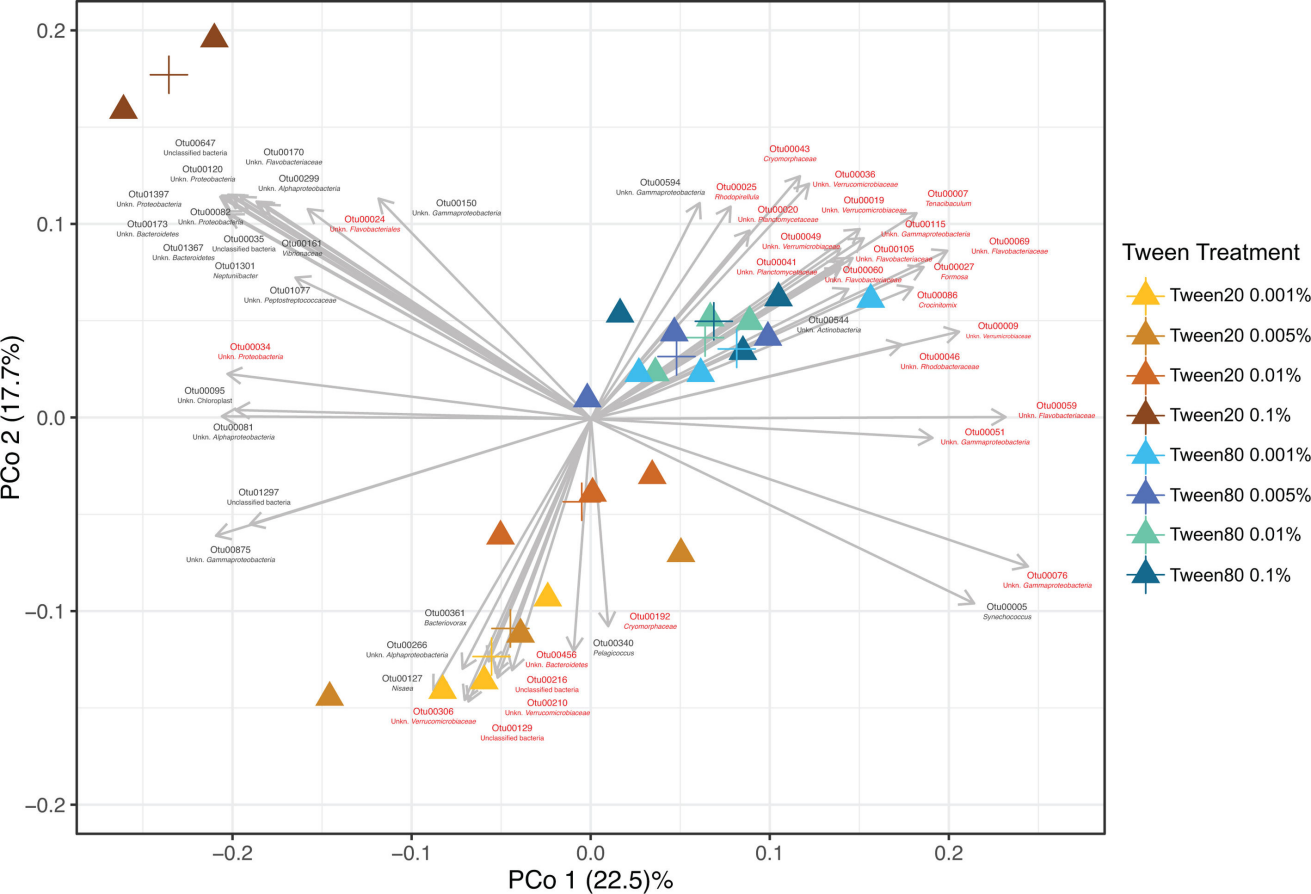
Principal coordinate analysis of the particle-associated communities that increased in the free-living fraction in response to Tween treatments (*P* value < 0.01). The color of the particle-associated (triangle) shapes corresponds to the Tween treatment, as indicated by the key. The crosses indicate the centroid points for each treatment. Vectors represent increased PA-OTUs with significant relationships (*P* value < 0.01) to the ordination axes, which are driving the differences in PA-OTU community composition between Tween treatments. Red vector labels represent E-PA-OTUs that increased significantly (*P* value < 0.05) in the FL fraction in response to at least one Tween treatment relative to controls (Fig. 6; Table S3).

Successful microbial dissociation from particles would result in these PA-OTUs becoming enriched in the FL fraction (0.22–2.7 µm). We calculated the fold change in PA-OTU relative abundance in the FL fraction after Tween treatments and identified 316 PA-OTUs that increased by more than onefold in at least one Tween treatment (Table S2). Using principal coordinate analysis of these 316 PA-OTUs, we found that distinct communities were liberated from Tween 80 and Tween 20 treatments, with the Tween 20 0.1% treatment diverging from all other treatments (Fig. 4; Fig. S3). OTUs liberated from Tween 80 treatments showed less variation in community composition compared to those liberated from Tween 20 treatments overall, implying a more consistent dissociation effect from Tween 80. The first two principal coordinates, PCo1 and PCo2, collectively explained only 40.2% of the variance in the community composition, which suggests that the differences in PA-OTU composition between Tween treatments were complex and the relationships were not easily reduced to the principal coordinates. Nevertheless, these results demonstrate that the choice of Tween treatment affects not only the magnitude of cellular dissociation (Fig. 1) but also the consistency of the enriched microbial community.

**Fig 5:**
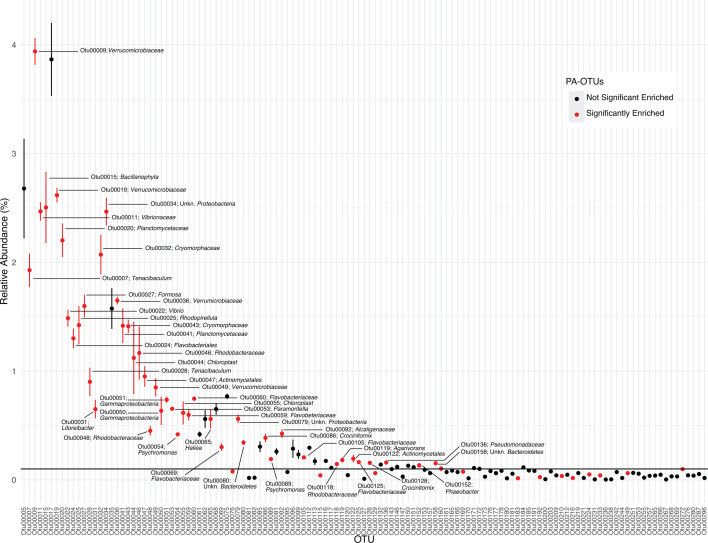
Rank abundance curve of the increased PA-OTUs and classification as abundant or rare. The *x*-axis shows individual OTUs in rank order, and the *y*-axis indicates their percent relative abundance. The black horizontal line shows a relative abundance of 0.1%. An OTU was considered “abundant” if it had a relative abundance > 0.1% in the control PA treatment. Datapoints and error bars represent the mean and variation in fold change in relative abundance across triplicates. Red data points indicate E-PA-OTUs that were significantly enriched in the FL-fraction in response to at least one Tween treatment (Fig. 6; Table S3). Data included are the top 125 increasing PA-OTUs.

**Fig 6:**
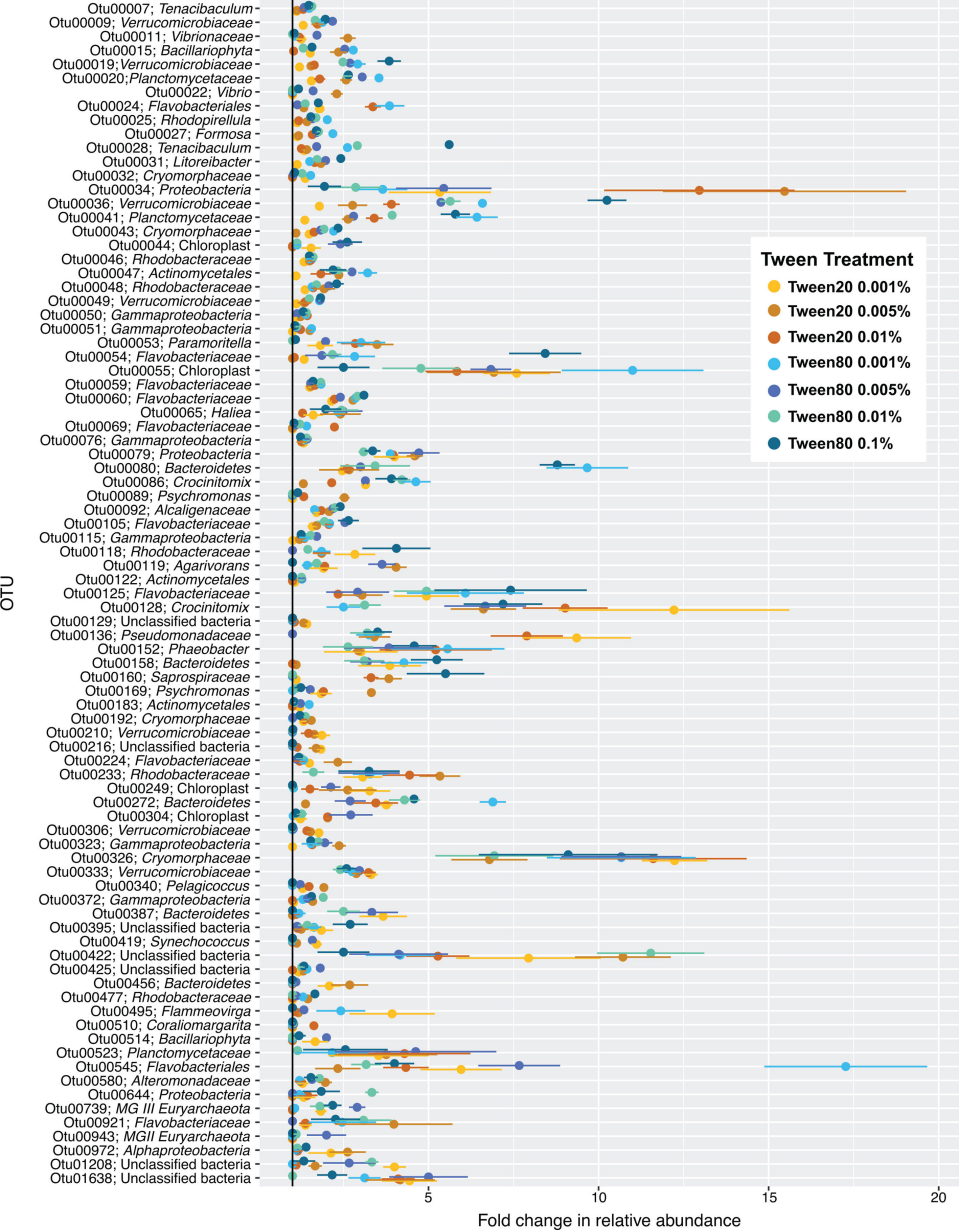
E-PA-OTU enrichment by Tween treatment. The E-PA-OTUs that significantly increased in at least one Tween treatment (Table S3) are organized by OTU number (from most overall relative abundance to least, top to bottom). The *x*-axis indicates the fold change in relative abundance, and the *y*-axis shows OTUs with their taxonomy. The color of the data points corresponds to the Tween treatment, as indicated by the key. Datapoints and error bars represent the mean and the range of variation in fold change in relative abundance across triplicates.

We observed that different PA-OTUs drove the separation between Tween 20 and Tween 80 communities, and that these differences were not associated with taxonomy (Fig. 4; Fig. S3 and Table S2). For example, PA-OTUs that were highly correlated with Tween 80 treatments included members from the phyla Proteobacteria, Bacteroidetes*,* Verrucomicrobia, Actinobacteria, and Planctomycetes, but we also observed this broad taxonomic representation in PA-OTUs driving variation across Tween 20 treatments (Fig. 4; Fig. S3). Instead of a taxonomic effect, we observed that OTU abundance was associated with the differences in enrichment by either Tween 20 or Tween 80. OTU abundance corresponds to OTU number (00001 being most abundant), and many of the most abundant PA-OTUs corresponded with the Tween 80 treatments in the principal coordinate analysis, whereas the PA-OTUs correlated with the Tween 20 treatments were less abundant or rare taxa, except for two OTUs associated with the Tween 20 0.1% treatment (Fig. 4; Fig. S3). When using a 0.1% relative abundance cutoff to define OTUs as “abundant,” of the 65 PA-OTUs at or above that cutoff, 21 of them were correlated with Tween 80 treatments and only three with Tween 20 treatments (Fig. 5; Fig. S3 and S4). Additionally, we defined enriched PA-OTUs (E-PA-OTUs) as those that significantly increased in at least one Tween treatment relative to the FL control (*n* = 85, *P* value ≤ 0.05, Fig. 6; Table S3). The majority of PA-OTUs that correlated with Tween 80 treatments in the principal coordinate analysis consisted of E-PA-OTUs, compared to Tween 20 treatments that corresponded to relatively few E-PA-OTUs (Fig. 4; Fig. S3 and Table S3). Altogether, these results indicated that Tween 80 treatments enriched for taxa that best represented the PA microbial fraction.

### Abundant vs rare PA-OTU enrichment

The fact that more abundant E-PA-OTUs (*P* value ≤ 0.05, Table S3) were correlated with Tween 80 treatments than with Tween 20 (Fig. 4; Fig. S3) did not mean that these were never enriched by Tween 20. Rather, Tween 80 treatments enriched these OTUs more than Tween 20 (Fig. 6), similarly to how Tween 80 liberated more viable cells overall (Fig. 2; Fig. S1). For example, one of the most abundant E-PA-OTUs, Otu00009 (member of Verrucomicrobiaceae*,* a commonly detected PA group [8, 54–59]), was enriched across all Tween treatments, but the greatest change in relative abundance was in the Tween 80 treatments, particularly Tween 80 0.005%, where its abundance more than doubled (Fig. 5 and 6; Fig. S5, *P* value = 0.04). Other abundant E-PA-OTUs, Otu00019 and Otu00028, members of the Verrucomicrobiaceae and *Tenacibaculum* within the Flavobacteriaceae, respectively (also typical PA community groups [8, 60]), were slightly enriched in Tween 20 treatments but were much more enriched among Tween 80 treatments (Fig. 6). Upon visual (Fig. 6) and statistical inspection (Table S3), most of these taxa were more highly enriched in Tween 80 treatments compared to Tween 20 treatments (Fig. 6), particularly the taxa with numerical OTU identifiers less than Otu00050 (Fig. S5), thus corroborating PCoA results (Fig. 4; Fig. S3). Additionally, all of these abundant E-PA-OTUs matched taxa that have been previously identified in PA communities (3, 8, 54, 61–64), confirming the taxonomic enrichment trends that we detected.

Although the majority of abundant E-PA-OTUs became enriched in the Tween 80 treatments, there were a few that were correlated with and better enriched by the Tween 20 treatments instead. Two of these—Otu00024 and Otu00034, unclassified members of Flavobacteriales and Proteobacteria, respectively—were strongly correlated with Tween 20 treatments (Fig. 4; Fig. S3), primarily driven by the highly divergent Tween 20 0.1% treatment (Fig. 4; Fig. S6). Tween 20 at 0.1% caused average fold change increases of 11 and 27 for Otu00024 and Otu0034, respectively, almost double the fold change increases for these OTUs across all other treatments (Fig. S6). Otu00136 and Otu00169, members of Pseudomonadaceae and *Psychromonas* of the Gammaproteobacteria, were also correlated with Tween 20 treatments (Fig. S3) but were predominantly driven by Tween 20 0.001%, 0.005%, and 0.01% treatments (Fig. 6). Otu00136 was most highly enriched by Tween 20 0.001% and 0.01%, increased by approximately six- and ninefold in each treatment, respectively. Whereas Otu00169 increased approximately threefold in Tween 20 0.005%. The enrichment of these OTUs in the Tween 20 treatments may indicate that the way in which they adhere to particles, or the particulate matrix on which these organisms reside, was particularly responsive to Tween 20 dissolution.

Rare E-PA-OTUs included numerical OTU identifiers greater than Otu00184 (Fig. 5; Table S4; Fig. S4), and upon visual (Fig. 6) and statistical inspection (Table S3), most of these taxa were more highly enriched in Tween 20 than Tween 80 treatments (Fig. 6; Fig. S6). Otu00192, a member of Cryomorphaceae (a commonly detected lineage within PA communities [60, 65]) in the Flavobacteriia, was enriched by Tween 80 0.01% and other Tween 20 treatments, but most highly enriched by Tween 20 0.005% (*P* value = 0.01) (Fig. 6; Fig. S5). Otu00210, 00216, 00306, and 00456 were enriched by at least two Tween 20 treatments, with almost no enrichment by any Tween 80 treatments (Fig. 6; Fig. S5). These OTUs included unclassified members of the Verrucomicrobiaceae and Bacteroidetes*,* both of which are consistently detected in other PA microbial communities (3, 8, 54–58, 61, 63, 65). Otu00419, a *Synechococcus* representative, was correlated with the Tween 20 treatments (Fig. S5) and enriched in Tween 20 0.001% and 0.005% specifically (Fig. 6; Fig. S4). Although Otu00419 was also enriched by Tween 80 0.005% (Fig. 6; Fig. S5), the effect was greater in the Tween 20 0.005% treatment, indicating that the correlation of Otu00419 to Tween 20 treatments was based on subtle differences. *Synechococcus* cyanobacteria are commonly detected in the PA fraction (8, 11, 62, 64, 66) and form aggregates in coastal waters, thus making them representative of the particulate organic matter pool (67).

Overall, our observed PA communities were dominated by bacteria; archaeal OTUs (nOTUs = 48) only constituted 0.048% ± 0.002% of the total (https://doi.org/10.6084/m9.figshare.29565185.v2; Complete_OTU_table); however, two archaeal OTUs, Otu00739 and Otu00934, were still among the E-PA-OTUs (Fig. 6; Fig. S5). Tween 20 treatments generally performed poorly at dissociating archaeal OTUs, aside from Tween 20 0.001%, which enriched Otu00739. Of the Tween 80 treatments, Tween 80 0.005% performed best at enriching archaeal OTUs, nearly tripling the relative abundance of Otu00739 (*P* value = 0.02) and nearly doubling the relative abundance of Otu00934 (*P* value = 0.03) (Fig. 6; Fig. S6). Otu00934 and Otu00739 belonged to Marine Group II (99.6% identical) and Marine Group III (99.6% identical), respectively, via blastn alignment (68). Both Marine Group II and Marine Group III have been detected in PA fractions (69–71), further corroborating the significant increases in PA-OTUs that we are measuring.

We do not know the mechanism by which Tween 80 outperformed Tween 20 in enriching for the most abundant PA-OTUs, but we speculate that it stems from differences in the Tween 20 and Tween 80 side chains. Tween 20 and Tween 80 are both characterized as polysorbate nonionic detergents, and they vary in their fatty acid side chains (72): Tween 20 has a saturated 12-carbon (12:0) lauric acid side chain, whereas Tween 80 has an unsaturated 18-carbon (18:1) oleic acid side chain (73). In general, longer alkyl chains in fatty acids are more hydrophobic. Although double bonds in alkyl chains decrease hydrophobicity, oleic acid has almost twice the hydrophobicity value of lauric acid (74). Thus, the higher degree of hydrophobicity of Tween 80 may better disrupt the microbial adhesion strategies, such as stalks or pili (45), which are typically hydrophobic in nature (75–77).

### Non-specific E-PA-OTUs

Some of the E-PA-OTUs were not associated with any particular Tween treatments, but rather increased across all treatments (Fig. 4 and 6; Fig. S3). For example, Otu00333, an unclassified member of the Verrucomicrobiaceae, a group that is commonly detected in the PA fraction (8, 54–58), approximately tripled in relative abundance across all treatments (Fig. 6). Similarly, Otu00125 (unclassified Flavobacteriaceae), Otu00128 (*Crocinitiomix*), and Otu00326 (Cryomorphaceae), all members of Bacteroidetes that have been previously detected in PA communities (3, 8, 54, 60, 61, 63, 65), were also highly enriched across all treatments (Fig. 6). Otu00079, an unclassified member of Proteobacteria, and Otu00152, a *Phaeobacter* representative from within the Alphaproteobacteria (another group previously detected in the PA-fraction [9, 65, 78]), were evenly enriched across all Tween treatments as well. Otu00545, an unclassified member of the Flavobacteriales, was highly enriched across all treatments, averaging approximately two to sevenfold increases in relative abundance, except in Tween 80 0.001%, where it increased approximately 17-fold (*P* value = 0.04) (Fig. 6). Despite being so highly enriched in Tween 80 0.001%, Otu00545 was not included among the OTUs that significantly drove differences in PA-OTUs among Tween treatments (Fig. 4; Fig. S3), likely due to overlapping fold changes in relative abundance between Tween 20 and the other Tween 80 treatments.

### Eukaryotic algal enrichment

The presence of chloroplasts among E-PA-OTUs (Fig. 6) was likely evidence of cell lysis, as most eukaryotic phytoplankton will not pass through a 2.7 µm pore-size filter. We identified unclassified chloroplast OTUs Otu00044, Otu00055, Otu00249, and Otu00304 as belonging to *Heterosigma akashiwo* (99.2% identical), *Fibrocapsa japonica* (100% identical), *Aureococcus anophagefferens* (99.6% identical), and an unknown marine eukaryote (99.21% identical), respectively. The first three are eukaryotic algae with cell sizes typically ranging from 3 to 60 µm (79–83). We also observed diatom chloroplast OTUs, Otu00015 and Otu00514, which corresponded to *Skeletonema pseudocostatum* (98.8% identical) and *Bolidophyceae* spp. (99.6% identical), respectively. *Skeletonema pseudocostatum* is a chain-forming diatom with individual cell sizes ranging from 2 to 9 µm (84, 85), and *Bolidophyceae* are algae within the Stramenopiles, sister to diatoms, with cell sizes ranging from 4 to 6 µm (86). These large cell sizes supported our hypothesis of eukaryotic cell lysis and/or chain disruption by the Tween treatments. One might assume that higher concentrations of detergent would lead to more cell lysis, but instead, we observed an overlap in eukaryotic chloroplast enrichment across the highest and lowest Tween concentrations (Fig. 6). Furthermore, our flow cytometry observations did not indicate that one treatment was causing increased cell mortality over another (Fig. 1; Fig. S2), even though the sample core on our flow cytometer was 16 µm in diameter, making it possible for us to detect compromised larger cells with the live/dead stain. This suggests that Tween treatments do disrupt eukaryotic cells, but the interactions are difficult to predict with the present data.

### Conclusion

Together, flow cytometry and community composition analyses indicated that Tween 20 and Tween 80 both effectively dissociate microbes from particles, but with different outcomes (Fig. 2, 4, and 6). Tween 80 treatments often dissociated a higher magnitude of PA cells, reaching upwards of a 70% increase in cell density (Fig. 2). Also, the cell mortality between Tween 20 and Tween 80 treatments was consistently similar, indicating that Tween 80 was just as gentle, but more effective than Tween 20. Additionally, shaking at 185 RPM was a more reliable perturbation method compared with vortexing, particularly for durations longer than 5 minutes (Fig. S2B). At the OTU level, Tween 80 demonstrated greater uniformity in the dissociated communities than Tween 20 (Fig. 4) and significantly enriched the most abundant PA-OTUs, thus capturing the members that best represented the PA community. Tween 80 treatments were also more effective in dissociating archaeal PA cells into the FL-fraction (Fig. 6; Fig. S5), particularly Tween 80 0.005% (Fig. 6). We recommend sampling for the FL and PA communities separately, as the Tween treatment does affect the community composition of the FL community (Fig. 3). Tween 80 also reliably and effectively dissociated a broad range of PA cells into the FL fraction across marine environments (Fig. 2, 4, and 5; Fig. S3 and S4). To limit the amount of detergent added to invaluable samples and capture a broad taxonomic representation of PA microbes with limited cell mortality, we recommend Tween 80 0.005%, as it strikes a balance between a gentle dissociation without compromising dissociative power.

## MATERIALS AND METHODS

### Environmental sample collection

For the 16S rRNA gene community composition analysis, we sampled 60 L of seawater on 20 November 2021 from the Santa Monica Bay (33.99675°N, 118.48603°W) via manual surface water collection into four 20-L sterile polycarbonate carboys with 5 L of headspace per carboy (Thermo Fisher Scientific, Waltham, MA, USA). Sample processing began within 1 hour of sample collection. For flow cytometry analyses, subsequent sampling from the same Santa Monica Bay site occurred in 2023 on 16 February (winter), 3 April (spring), 29 June (summer), and 26 November (fall), with 3 L of surface seawater collected into sterile 4-L carboys (Thermo Fisher Scientific, Waltham, MA, USA), and sample processing beginning within 1 hour of sample collection. For additional flow cytometry analysis, samples were collected on 15 May 2024 from the San Pedro Ocean Time-series sampling site (33.55°N, 118.4°W) to reflect microbial communities that are more characteristic of offshore waters (87) with offshore geochemistry (88). Seven liters of surface seawater (2 m) was collected via Niskin bottles and transferred into a sterile 10-L carboy (Thermo Fisher Scientific, Waltham, MA, USA). Due to the logistics of sample collection, the carboy was stored at close to ambient seawater temperature (20°C), and sample processing was performed within 18 hours of sample collection.

### Microbial dissociation from particles

We tested four concentrations of Tween 20 (Sigma-Aldrich, St. Louis, MO, USA) and Tween 80 (Sigma-Aldrich, St. Louis, MO, USA). We diluted stock solutions of 5% Tween 20 and Tween 80 (vol/vol) in sterile phosphate-buffered saline (1×) into surface seawater (sample volumes below) at final concentrations of 0.001%, 0.005%, 0.01%, and 0.1% (vol/vol), along with untreated controls. We separated particle-associated and free-living taxa via size fractionation, where cells caught on a 2.7 µm pore size GF/D filter (Whatman, Maidstone, UK) were considered particle-associated, while cells that flowed through the 2.7 µm filter were considered planktonic/free-living. The logic of the experimental design was that PA cells that were previously collected on 2.7 µm filters would pass through these filters after treatment with the detergent and dissociation from particles. Thus, after dissociation, formerly PA cells would be detected in the 2.7 µm filtrate either via flow cytometry or by collecting them on smaller filter pore sizes (0.22 µm). The relative success of the detergent would be quantified via the relative increase in total cells (evaluated via propidium iodide vs SYBR Green staining, see below) and/or PA community members in the 2.7 µm filtrate.

For flow cytometry analyses, we diluted Tween 20 and Tween 80 into sterile 50 mL Falcon tubes (VWR, West Chester, PA, USA) containing 30 mL surface seawater from the Santa Monica Bay and SPOT at the concentrations described above in triplicate. Samples were either vortexed at maximum speed for 5 or 10 minutes, or they were shaken at 185 RPM for 5 or 10 minutes. For vortexing at maximum speed, the sample tubes were placed in a vertical/upright position on the vortexer. For 185 RPM shaking, the sample tubes were secured in a horizontal position to increase the surface area for better mixing. After vortexing/shaking, we syringe-filtered the Tween-treated seawater through a sterile 2.7 µm 25 mm GF/D filter (Whatman, Maidstone, UK) and collected the filtrate in a new sterile 50 mL Falcon tube. Each replicate received a new 50 mL sterile syringe (VWR, West Chester, PA, USA) and a sterile 25 mm GF/D filter. We processed control samples in the same manner; however, these samples received no Tween treatment and no physical perturbation. To understand the effect that the perturbation alone had on the microbial communities, we processed additional controls from the Santa Monica Bay winter and fall samples, along with the SPOT samples, without Tween but with both vortexing and shaking, separately, for 5 and 10 minutes (Fig. 2; Fig. S2). We quantified cell concentrations from aliquots of the 2.7 µm filtrate for each treatment and replicate with a BD Accuri C6 Plus flow cytometer (BD, New Jersey, USA) after a 30-minute 1× SYBR Green (Lonza Bioscience, Basel, Switzerland) dark, room-temperature incubation. Separately, the number of dead cells was also quantified from aliquots of the 2.7 µm filtrate from each treatment and replicate using the same flow cytometer after a 30-minute 1× propidium iodide (Thermo Fisher Scientific, Waltham, MA, USA) dark, room-temperature incubation. Results were visualized in R version 4.4.0 (89, 90) using ggplot2 (91). We then used the Wilcoxon signed-rank test (92) in R version 4.4.0 (89, 90) to evaluate which Tween treatments were significantly different relative to the control treatment that received physical perturbations.

For 16S rRNA gene community composition analysis, we diluted the four different concentrations of Tween 20 and Tween 80 into separate, sterilized 10-L carboys containing 7 L of surface seawater from the Santa Monica Bay in triplicate. We manually shook each carboy for 5 minutes. The free-living (Sterivex) Tween 20 0.1% only received duplicate samples. Subsequently, 2 L of the Tween-treated seawater was filtered via peristaltic pumping (Cole Parmer 77601-10, Vernon Hills, IL, USA) with an in-line (Masterflex I/P tubing, Germany) sterile 2.7 µm 47 mm GF/D filter (Whatman, Maidstone, UK) and a sterile 0.22 µm Sterivex filter (Sigma-Aldrich, St. Louis, MO, USA). Each replicate received new sterile filters, and filter lines were flushed with 1 L of 70% ethanol and 2 L MilliQ water between samples. Control samples that received no physical perturbation and no Tween treatment were also filtered in triplicate through the same in-line filtration setup. Prior to filtration, filter lines were sterilized with equal volumes of 0.1 N HCl (Sigma-Aldrich, St. Louis, MO, USA), 0.1 N NaOH (Sigma-Aldrich, St. Louis, MO, USA), and MilliQ water. After filtration, samples were immediately stored at −20°C until further processing.

### DNA extraction and sequencing

We extracted DNA for 16S rRNA gene sequencing from all GF/D and Sterivex filters across all treatments based on a phenol-chloroform-isoamyl alcohol extraction method and the Griffiths Method (93). Briefly, we removed filters from −20°C storage and thawed them on ice. We tore GF/D filters into quarters using sterile forceps and placed each quarter into a sterile microcentrifuge tube. We sliced Sterivex filters in half using sterile razor blades and placed each half into a sterile microcentrifuge tube with sterile forceps. Chemical lysis was performed by adding 557 µL 1× Tris-EDTA buffer (Sigma-Aldrich, St. Louis, MO, USA), 139 µL 10% SDS (Sigma-Aldrich, St. Louis, MO, USA), and 35 µL lysozyme (1.4 mg/mL final concentration, Sigma-Aldrich, St. Louis, MO, USA) to each tube and vortexing for 30 seconds, then incubating at 37°C for 30 minutes. After incubation, we added 70 µL of proteinase K (0.7 mg/mL final concentration, Sigma-Aldrich, St. Louis, MO, USA) to each tube, vortexed for 20 seconds, and then incubated overnight at 55°C (94). After overnight incubation, we separated the lysate from the GF/D filters by transferring the GF/D filter + lysate into sterile 0.45 µm pore size Costar Spin-X tubes (CLS8162, Sigma-Aldrich, St. Louis, MO, USA) and centrifuged at 12,000 × *g* for 1 minute. Then, the lysate from the GF/D filter was transferred into a new sterile microcentrifuge tube. Lysate separation for the Sterivex filter was unnecessary, as the polyethersulfone membrane is dissolved during extraction. After overnight lysis incubation and lysate separation from GF/D filters, we added 500 µL of CTAB extraction buffer (93) and 500 µL of phenol-chloroform-isoamyl alcohol (25:24:1, pH 8.0, Sigma-Aldrich, St. Louis, MO, USA) to the lysate, vortexed for 30 seconds, then centrifuged at 16,000 × *g* at 4°C for 5 minutes. We transferred the aqueous layer to a new sterile microcentrifuge tube, and an equal volume of chloroform-isoamyl alcohol (24:1, Sigma-Aldrich, St. Louis, MO) was added, then centrifuged again at 16,000 × *g* at 4°C for 5 minutes. We again transferred the aqueous layer to a new sterile microcentrifuge tube and precipitated the DNA for 1 hour at room temperature with a 0.1× aqueous volume 3 M sodium acetate (pH 5.2, Sigma-Aldrich, St. Louis, MO, USA) and 0.6× aqueous volume isopropanol (95), then centrifuged at 16,000 × *g* at 4°C for 15 minutes. We washed the DNA pellet with 150 µL of ice-cold 100% (vol/vol) molecular grade ethanol (Sigma-Aldrich, St. Louis, MO, USA) and centrifuged again at 16,000 × *g* at 4°C for 15 minutes, air dried, and resuspended in 50 µL of molecular-grade water. Pure DNA was achieved by incubating resuspended DNA with RNase A (Promega, Madison, WI, USA) for 10 minutes according to the manufacturer’s instructions. Purified DNA was stored at −80°C until sequencing.

Prokaryotic genomic DNA was amplified at the V4 region of the 16S rRNA gene using the 515F and the modified 806R primer set (96, 97) and QuantaBio’s AccuStart II PCR ToughMix (QuantaBio, Beverly, MA, USA). Amplicons were sequenced at Argonne National Laboratory on an Illumina MiSeq using paired-end 250 bp reads using the version 2.5 TruSeq Paired End MiSeq flow cell/cluster kit (Illumina Inc., San Diego, CA, USA) according to the manufacturer’s instructions (96).

### Community composition

To assess how prokaryotic community composition changed in response to Tween treatments, raw 16S rRNA gene sequences were analyzed using Mothur version 1.48.0 (98) and the Silva version 132 database (99), keeping replicates separate. We first assembled 16S rRNA gene amplicon sequences into contigs and discarded contigs with ambiguous bases, nucleotide repeats greater than 8 bp, or those that were greater than 275 bp in length. We aligned and classified contigs via the Silva version 132 database and removed chimeric contigs with Mothur version 1.48.0. Contigs that were classified as “unknown” were removed, and the remaining contigs were clustered into OTUs with a 0.03 dissimilarity threshold, resulting in 30,258 OTUs with a mean length of 252 bp.

To assess the overall dissimilarity in OTU composition between size fractions and Tween treatments, we calculated Bray-Curtis dissimilarity (100) distance matrices and applied them toward PCoA (101) in Mothur version 1.48.0 (91). We quantified the distribution of OTUs across technical replicates for all treatments in R version 4.4.0 using VennDiagram (102). A PA-OTU was defined as such if it was present across all three GF/D filters and vice versa for the FL-OTUs from control Sterivex filter replicates. To determine if PA-OTUs were becoming enriched in the FL fraction after Tween treatment, first, we calculated fold changes in the relative abundance of PA-OTUs across all Tween treatments in the FL fraction. A PA-OTU with a fold change in relative abundance greater than one in the FL fraction was considered to have increased. We also defined an increased PA-OTU as “abundant” if it had a relative abundance of ≥0.1% in the control PA community, while “rare” increased PA-OTUs were those with <0.1% relative abundance in the control PA community. Second, focusing on the PA-OTUs with more than onefold increased abundance, we calculated Bray-Curtis dissimilarity (100) distance matrices and performed a PCoA (101) on this group (nOTU = 316). Third, we fitted vectors for significantly increased PA-OTUs onto the ordination using the “envfit” function from the Vegan (103) package. Fourth, we then used the Wilcoxon signed-rank test (92) in R version 4.4.0 (89, 90) to evaluate which of the increased PA-OTUs were significantly enriched in each of the Tween treatments relative to controls (E-PA-OTUs) and visualized those results in R version 4.4.0 (89, 90) using ggplot2 (91). If an OTU within the E-PA-OTUs was classified as “unclassified bacteria,” we attempted to improve the taxonomic classification manually by aligning the OTU consensus sequence to the NCBI *nr* database (104) via BLASTN (68).

## Data Availability

Raw sequencing fastq files are available on NCBI under BioProject PRJNA1134785. Accessory data, including a complete OTU table and a file with the OTU consensus sequences, as well as scripts used for 16S rRNA gene analyses, statistical tests, and data visualization, are available on FigShare (https://doi.org/10.6084/m9.figshare.29565185.v2).

## SUPPLEMENTAL MATERIAL

Figure S1 aem.01426-25-s0001.pdf Additional flow cytometry data.

Figure S2 aem.01426-25-s0002.pdf Percentage of OTUs distributed across replicates.

Figure S3 aem.01426-25-s0003.pdf PCA of the particle-associated communities that increased in the free-living fraction in response to Tween treatments (*P* < 0.05).

Figure S4 aem.01426-25-s0004.pdf Rank abundance curve of the increased PA-OTUs and classification as abundant or rare.

Figure S5 aem.01426-25-s0005.pdf E-PA-OTUs with fold change increases less than 3.5.

Figure S6 aem.01426-25-s0006.pdf E-PA-OTU enrichment by Tween treatment, including Tween20 0.1%.

Table S1 aem.01426-25-s0007.xlsx Statistical evaluation of cell density increases via flow cytometry.

Table S2 aem.01426-25-s0008.xlsx PA-OTUs that increased with fold-change greater than one in response to Tween treatments.

Table S3 aem.01426-25-s0009.xlsx Statistical enrichment of PA-OTUs.

Table S4 aem.01426-25-s0010.xlsx Classification of increased PA-OTUs as either abundant or rare.

ASM does not own the copyrights to Supplemental Material that may be linked to, or accessed through, an article. The authors have granted ASM a non-exclusive, world-wide license to publish the Supplemental Material files. Please contact the corresponding author directly for reuse.
