# Supplementary material for: Detergent-based separation of microbes from marine particles
Source: Appl Environ Microbiol. 2025 Sep 25;91(10):e01426-25. doi: 10.1128/aem.01426-25 (PMC12542791; doi:10.1128/aem.01426-25)
Supplement: Figure S1 — Additional flow cytometry data. [file aem.01426-25-s0001.pdf]

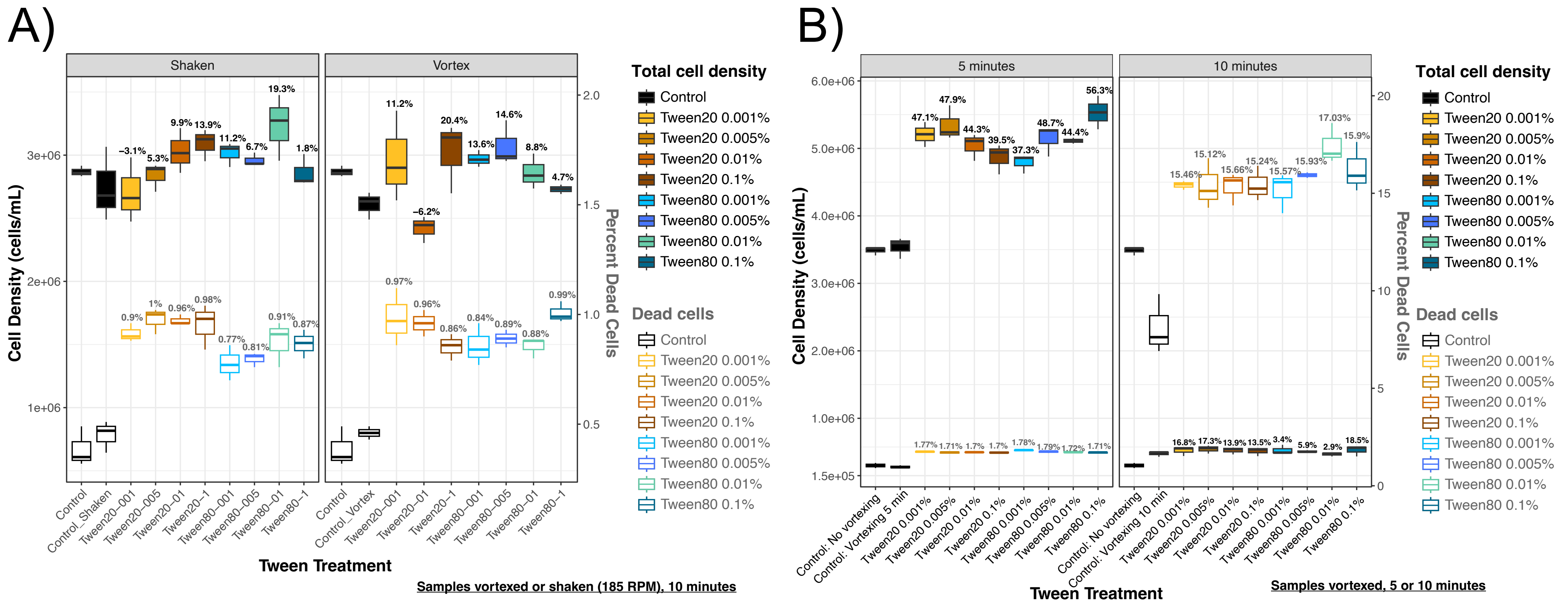

Figure S1. Additional flow cytometry data. X-axes indicate treatments. The left y-axis represents the cell density determined by the SYBR Green fluorescent stain (black text). The right y-axis represents the percentage of dead cells within the sample as determined by the Propidium Iodide fluorescent stain (grey text). The percentages above the boxplots indicate the average percent increase in cell density, or percent cell death, relative to the control. The boxplots describe the distribution of the data, with the boxes indicating the lower and upper quartiles, the horizontal line indicating the median, and the whiskers showing the minimum and maximum values. A) Samples collected from the Santa Monica Bay on June 29, 2023 (representing the summer season). Samples were either shaken at 185 RPM or vortexed, both for 10 minutes. The percentages above the box plots are derived from the control that also received either the shaking or vortexing perturbation treatment. B) Samples collected from the Santa Monica Bay on November 26, 2023 (representing the fall season). Samples were vortexed for either 5 minutes or 10 minutes. The percentages above the box plots are derived from the control that also received the vortexing perturbation treatment. Please note that due to the high cell mortality in the 10-minute treatment, the boxplots for “Cell Density” are below those for “Percent Dead Cells”, opposite to the other treatments.
