# Supplementary material for: Detergent-based separation of microbes from marine particles
Source: Appl Environ Microbiol. 2025 Sep 25;91(10):e01426-25. doi: 10.1128/aem.01426-25 (PMC12542791; doi:10.1128/aem.01426-25)
Supplement: Figure S2 — Percentage of OTUs distributed across replicates. [file aem.01426-25-s0002.pdf]

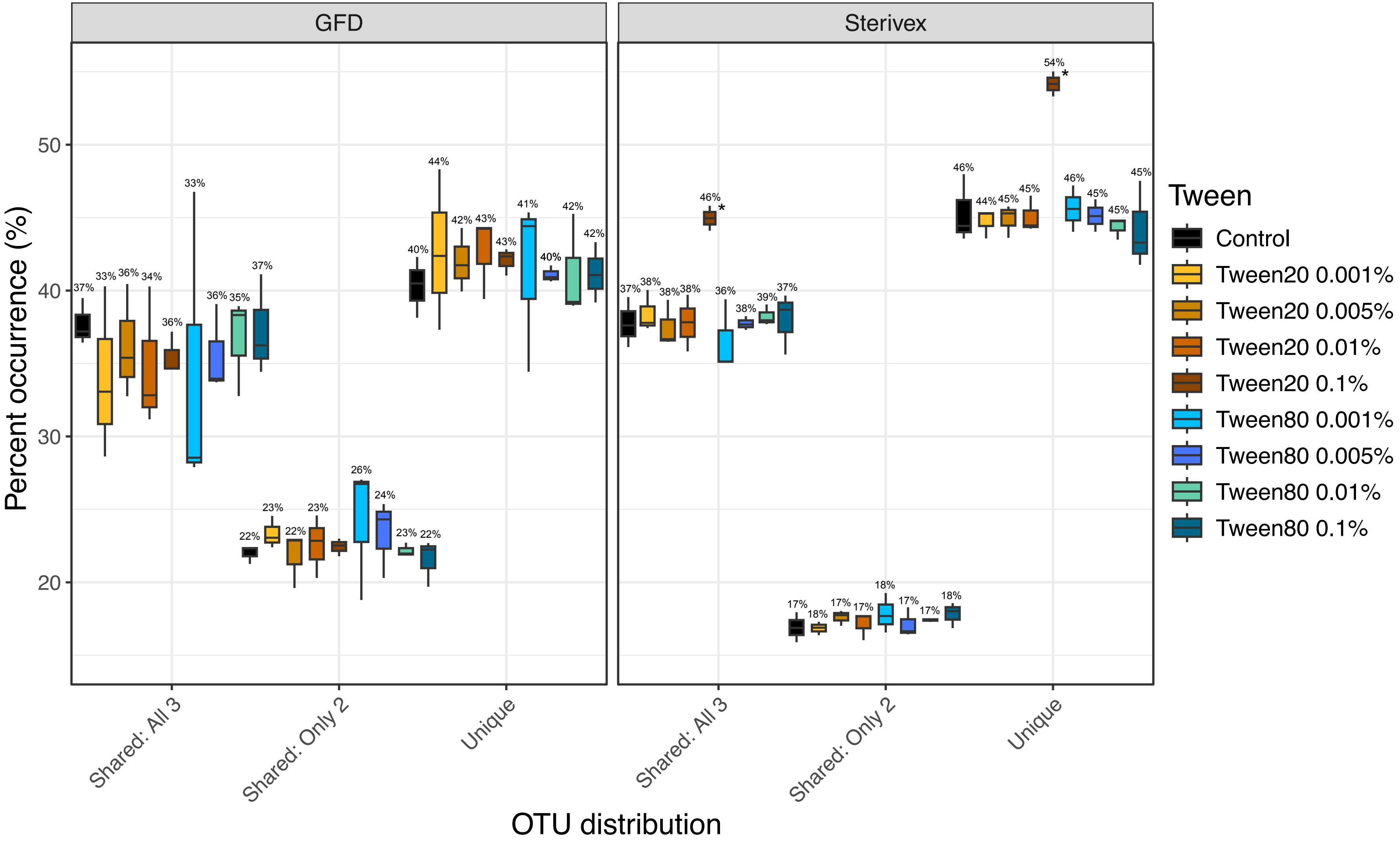

Figure S2. Percentage of OTUs distributed across replicates. X-axis indicates the OTU distribution category; y-axis indicates the percentage of OTUs in each category. The boxplots describe the distribution of the data, with the boxes indicating the lower and upper quartiles, the horizontal line indicating the median, and the whiskers showing the minimum and maximum values. The color of the boxplot corresponds to the control or experimental treatment, according to the key. Tween20 0.1% sterivex filters only received duplicate rather than triplicate replicates (*).
