# Supplementary material for: Detergent-based separation of microbes from marine particles
Source: Appl Environ Microbiol. 2025 Sep 25;91(10):e01426-25. doi: 10.1128/aem.01426-25 (PMC12542791; doi:10.1128/aem.01426-25)
Supplement: Figure S4 — Rank abundance curve of the increased PA-OTUs and classification as abundant or rare. [file aem.01426-25-s0004.pdf]

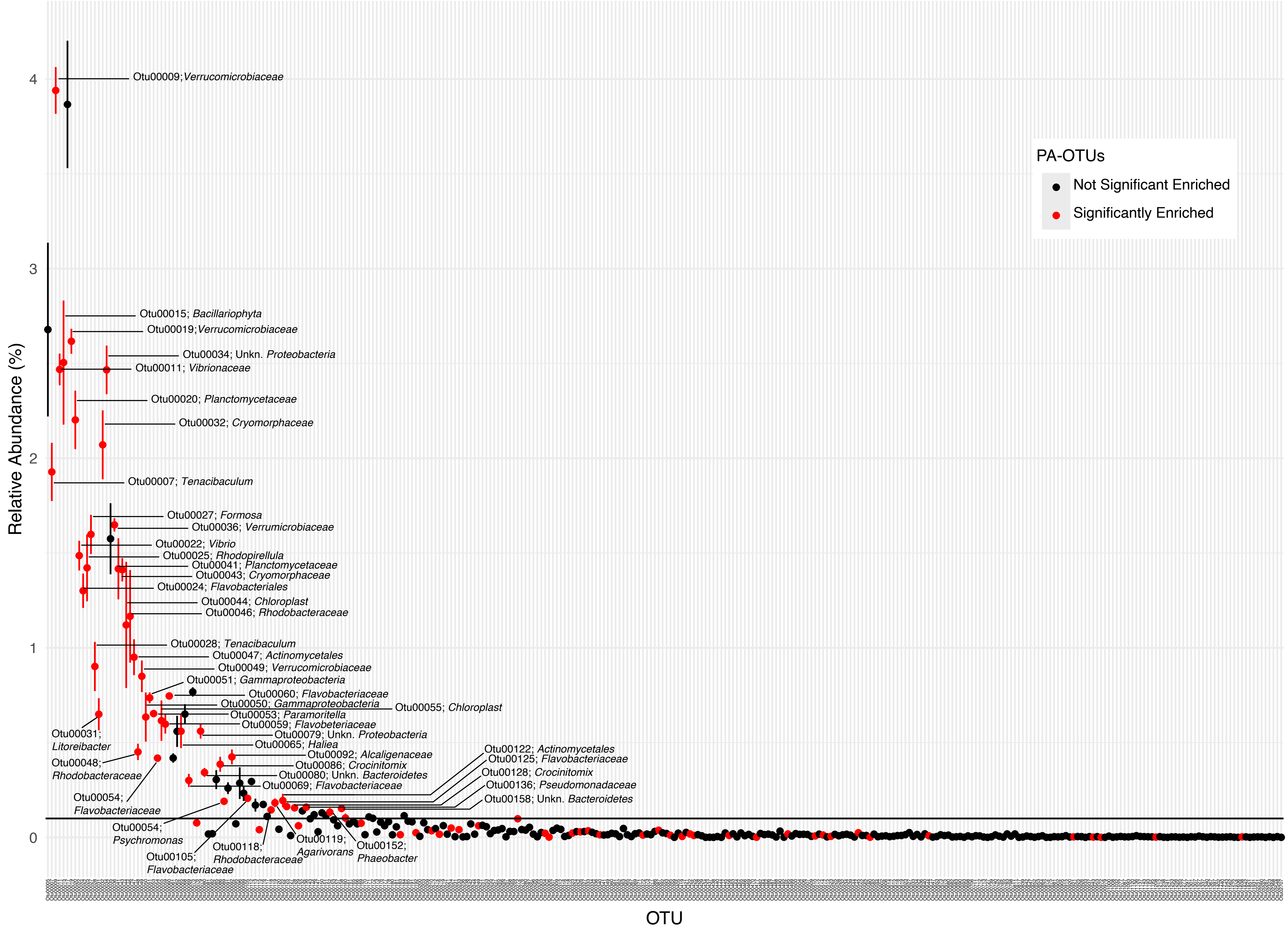

Figure S4. Rank abundance curve of the increased PA-OTUs and classification as abundant or rare. The x-axis shows individual OTUs in rank-order, and the y-axis indicates their percent relative abundance. The black horizontal line shows a relative abundance of 0.1%. An OTU was considered “abundant” if it had a relative abundance > 0.1% in the control PA treatment. Datapoints and error bars represent the mean and variation in fold-change in relative abundance across triplicates. Red data points indicate E-PA-OTUs that were significantly enriched in the FL-fraction in response to at least one Tween treatment (Figure 6, Table S3). Data are the same as Figure 5, but include all 316 increased PA-OTUs, instead of only the top 125.
