# Supplementary material for: Detergent-based separation of microbes from marine particles
Source: Appl Environ Microbiol. 2025 Sep 25;91(10):e01426-25. doi: 10.1128/aem.01426-25 (PMC12542791; doi:10.1128/aem.01426-25)
Supplement: Figure S5 — E-PA-OTUs with fold change increases less than 3.5. [file aem.01426-25-s0005.pdf]

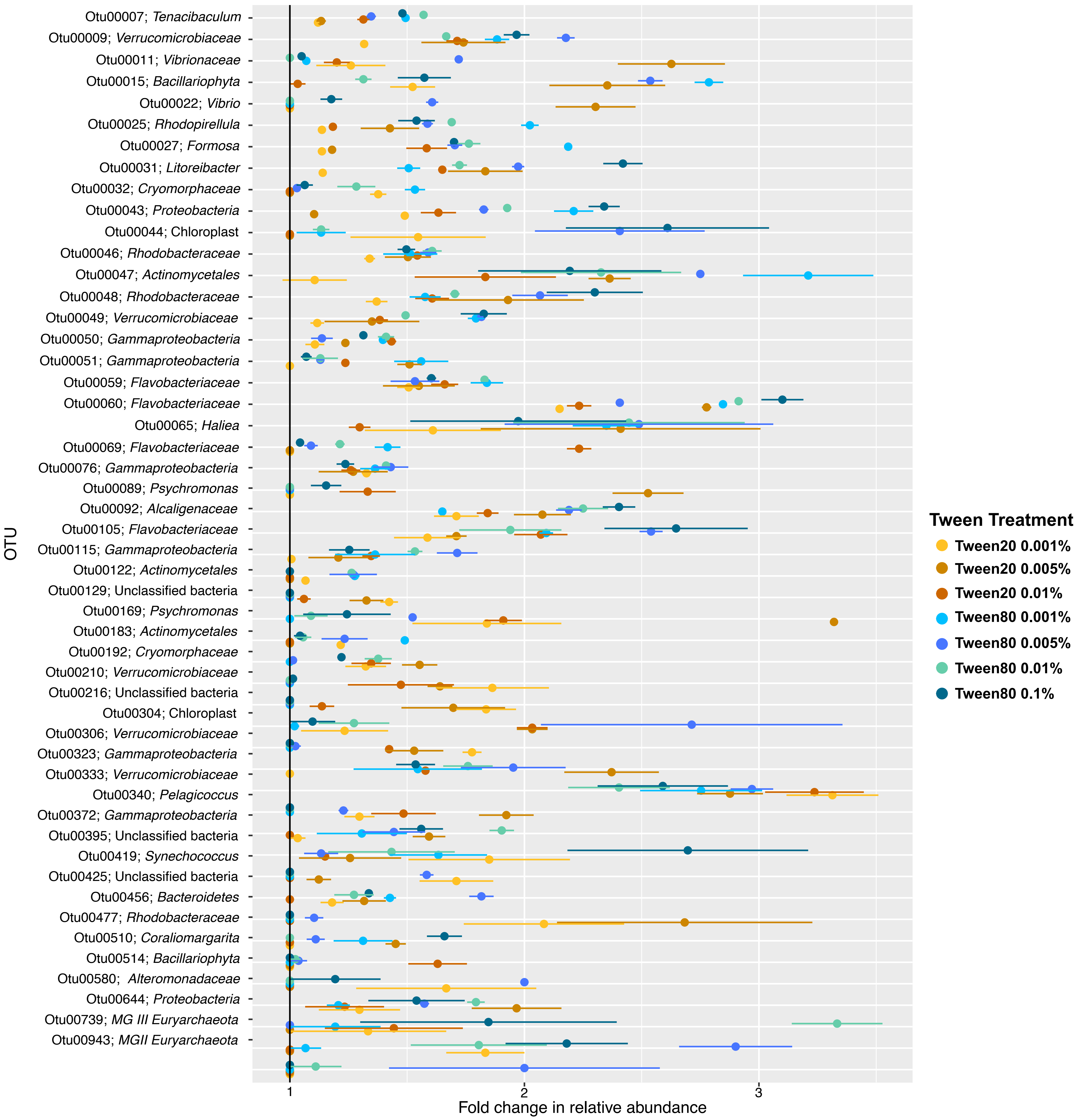

Figure S5. E-PA-OTUs with fold change increases less than 3.5. Same data as in Figure 6, subsetted for E-PA-OTUs with less enrichment to improve resolution. The E-PA-OTUs that significantly increased in at least one Tween treatment (Table S3) are organized by OTU number (most overall relative abundance to least). The x-axis indicates the fold change in relative abundance, and the y-axis shows OTUs with their taxonomy. The color of the data points corresponds to the Tween treatment, as indicated by the key. Datapoints and error bars represent the mean and variation in fold-change in relative abundance across triplicates.
