# Supplementary material for: Detergent-based separation of microbes from marine particles
Source: Appl Environ Microbiol. 2025 Sep 25;91(10):e01426-25. doi: 10.1128/aem.01426-25 (PMC12542791; doi:10.1128/aem.01426-25)
Supplement: Figure S6 — E-PA-OTU enrichment by Tween treatment, including Tween20 0.1%. [file aem.01426-25-s0006.pdf]

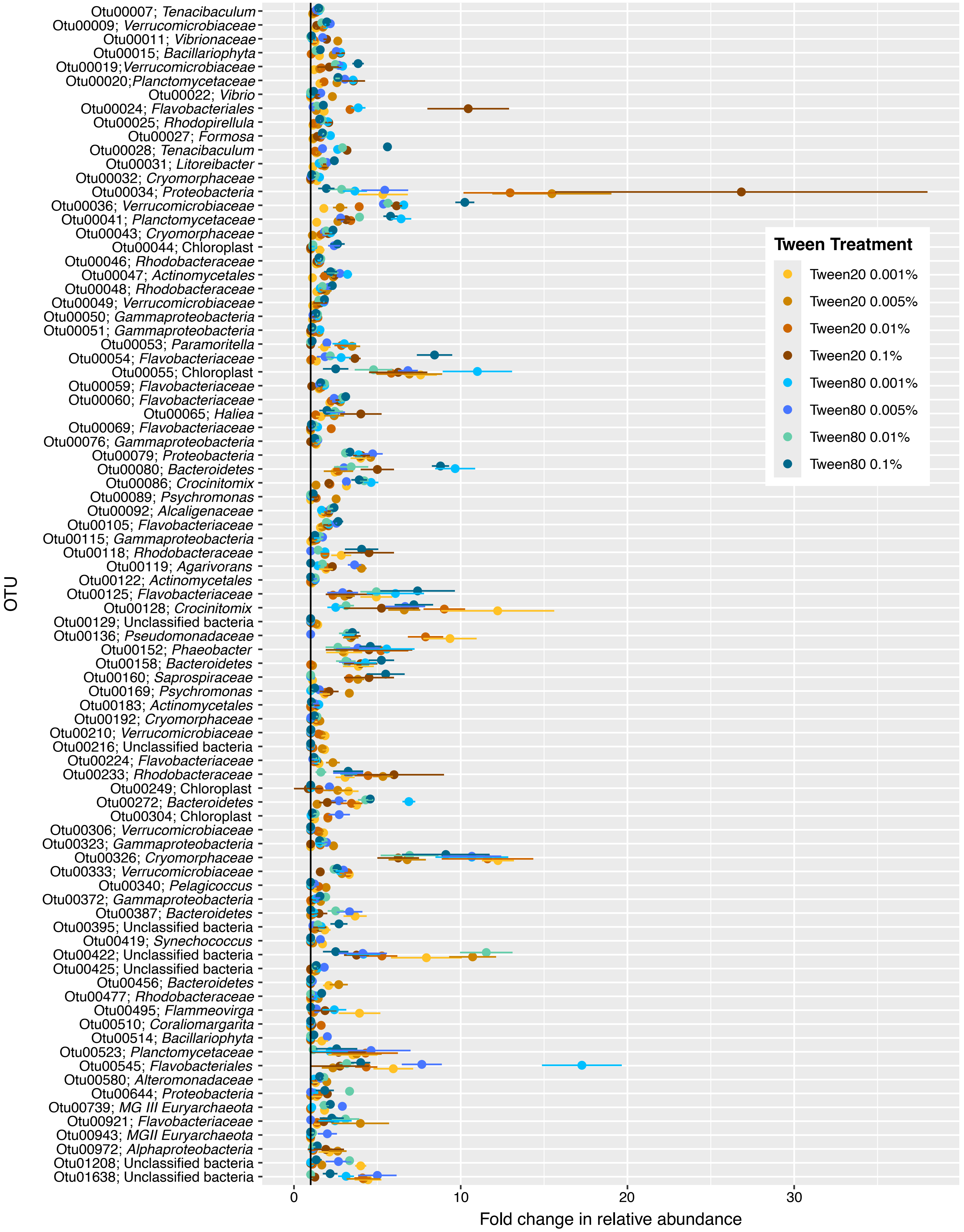

Figure S6. E-PA-OTU enrichment by Tween treatment, including Tween20 0.1%. The E-PA-OTUs that significantly increased in at least one Tween treatment (Table S3) are organized by OTU number (most overall relative abundance to least). The x-axis indicates the fold change in relative abundance, and the y-axis shows OTUs with their taxonomy. The color of the data points corresponds to the Tween treatment, as indicated by the key.
