# Supplementary material for: Detergent-based separation of microbes from marine particles
Source: Appl Environ Microbiol. 2025 Sep 25;91(10):e01426-25. doi: 10.1128/aem.01426-25 (PMC12542791; doi:10.1128/aem.01426-25)
Supplement: Figure S3 — PCA of the particle-associated communities that increased in the free-living fraction in response to Tween treatments (P < 0.05). [file aem.01426-25-s0003.pdf]

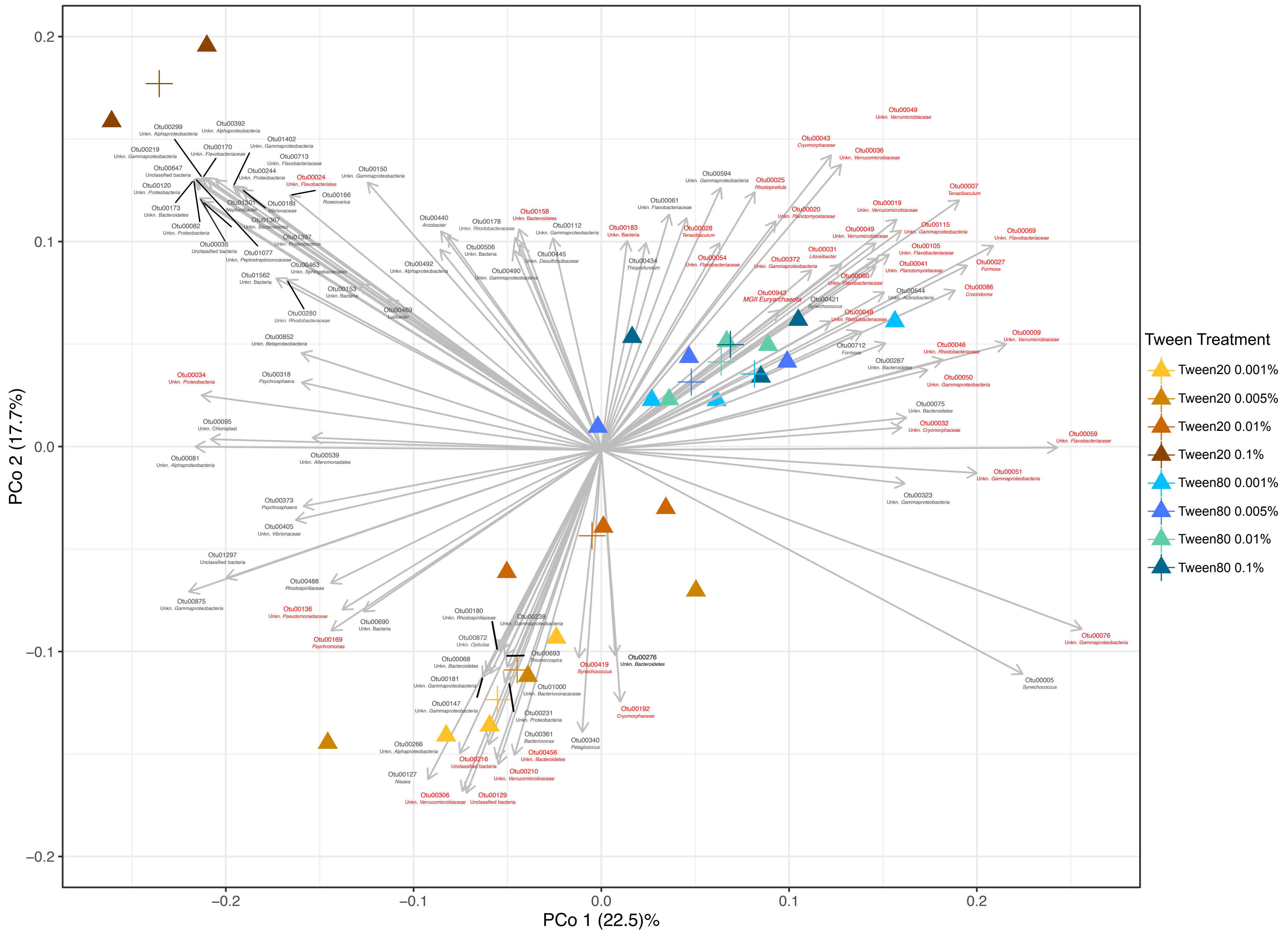

Figure S3. Principal Coordinate Analysis of the particle-associated communities that increased in the free-living fraction in response to Tween treatments (P value < 0.05). The color of the particle-associated triangles corresponds to the Tween treatment, as indicated by the key. The crosses indicate the centroid points for each treatment. Vectors represent OTUs with significant relationships (P value < 0.05) with ordination axes. Red vector labels represent an OTU that significantly increased in the FL fraction in response to at least one Tween treatment (Figure 6, Table S3).
